# Tick-borne pathogens in golden jackals (*Canis aureus*) from Armenia

**DOI:** 10.1186/s13071-026-07521-y

**Published:** 2026-07-25

**Authors:** Seda Adamyan, Margarida Ruivo, Iuliia Nosulia, Oleg Shcherbakov, Hasmik Gevorgyan, Sargis A. Aghayan, Julia Walochnik, Michiel Wijnveld

**Affiliations:** 1 Molecular Parasitology, Institute for Specific Prophylaxis and Tropical Medicine, Center for Pathophysiology, Infectiology and Immunology, Medical University of Vienna, Vienna, Austria; 2 Institute for Hygiene and Applied Immunology, Center for Pathophysiology, Infectiology and Immunology, Medical University of Vienna, Vienna, Austria; 3 Research Institute of Wildlife Ecology, University of Veterinary Medicine, Vienna, Austria; 4 Laboratory of Molecular Parasitology, Scientific Center of Zoology and Hydroecology, Yerevan, Armenia; 5 Research Institute of Biology, Yerevan State University, Yerevan, Armenia

**Keywords:** Tick-borne pathogens, Golden jackals, Canids, Wildlife, Transcaucasia, One Health

## Abstract

**Background:**

Golden jackals (*Canis aureus*) are expanding their range across Europe and Western Asia, frequently inhabiting agricultural and peri-urban areas where they interface with humans and domestic animals.

**Purpose:**

The aim of this study was to assess their potential role as reservoirs for tick-borne pathogens in Armenia, Transcaucasia.

**Methods:**

Between August 2024 and March 2026, blood samples were collected from 77 golden jackals across six administrative regions of Armenia (Lori, Tavush, Armavir, Ararat, Vayots Dzor, and Syunik). PCR followed by reverse line blot (RLB) hybridization was employed to simultaneously screen for bacterial and protistan pathogens, including *Anaplasma, Borrelia*, *Ehrlichia*, and *Rickettsia*, and *Babesia* and *Theileria* species, respectively.

**Results:**

Six zoonotic pathogens were detected: *Rickettsia helvetica* (16.9%), *Borrelia burgdorferi* sensu stricto (3.9%), *Babesia canis* (3.9%), *Rickettsia raoultii* (2.6%), *Candidatus* Neoehrlichia mikurensis (2.6%), and *Borrelia afzelii* (1.3%). Co-infections were detected in 7.8% of all samples (35.3% of pathogen-positive samples), including one triple co-infection with *R. helvetica*, *B. burgdorferi* s.s., and *Ca.* N. mikurensis*.* Statistically significant associations were observed between *R. helvetica* and *B. burgdorferi* s.s. (*r*_tet_ = 0.915) and between *R. helvetica* and *R. raoultii* (*r*_tet_ = 0.878). *Rickettsia helvetica* prevalence was significantly higher in females than in males (27.8% vs 7.3%; *p* = 0.030). Age-related trends suggested higher overall infection rates in adults (32.3%) compared to juveniles (15.2%; *p* = 0.097).

**Conclusions:**

This study provides the first molecular evidence of tick-borne pathogens in golden jackals from Transcaucasia, highlighting them as potential reservoir hosts for zoonotic pathogens with One Health significance.

**Graphical Abstract:**

**Figure:**
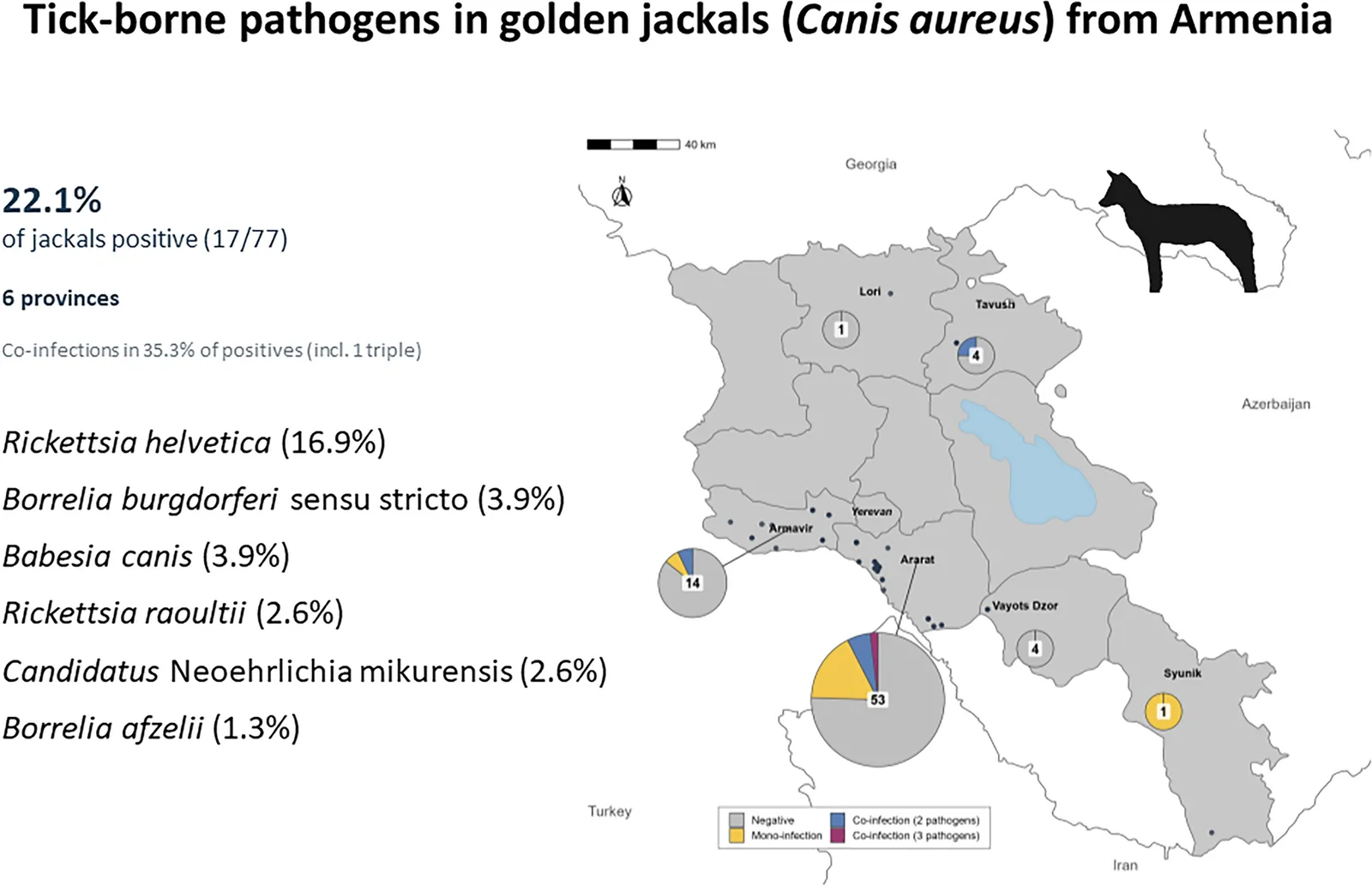

## Background

Tick-borne pathogens (TBPs) have a significant impact on global health. They include viruses (e.g., tick-borne encephalitis virus), bacteria (e.g., *Borrelia* spp., *Rickettsia* spp., *Anaplasma* spp., *Ehrlichia* spp.), and protists (e.g., *Babesia* spp., *Theileria* spp.*, Hepatozoon* spp.) and affect the human population, livestock, and wildlife worldwide [1, 2]. The circulation of these pathogens is through complex multi-host transmission patterns mediated by ixodid and argasid ticks [3]. The distribution of TBPs is amplified by the feeding ecology of ticks, which typically feed on taxonomically diverse blood host species with at least one blood meal in every developmental stage, facilitating horizontal pathogen transmission and resulting in individual ticks harboring multiple pathogens simultaneously [4, 5]. Some pathogens are also transmitted vertically from female ticks to their offspring, so tick larvae can already be infected. Climate change and anthropogenic landscape modification are driving the range expansion of tick vectors and vertebrate hosts, creating new interfaces for pathogen transmission and amplifying the risk of zoonotic transmission [6, 7].

In the Caucasus region, a high prevalence of TBPs has recently been documented in Armenian tick populations with an overall infection rate of 64%, dominated by *Anaplasma phagocytophilum* and *Theileria* spp. [8]. Additionally, Crimean-Congo hemorrhagic fever has been detected in ixodid ticks across multiple Armenian provinces [9–11], and tularemia remains endemic in southeastern Armenia [12]. However, substantial knowledge gaps remain regarding the reservoir hosts, particularly the role of wildlife species in the circulation of TBPs in this region.

Wild carnivores occupy a critical position in TBP ecology due to their role as bridge species connecting wild and domestic transmission cycles [13, 14]. Their broad spatial movements across habitat types, combined with infestation by tick species that parasitize both wildlife and livestock, make them sentinel indicators of pathogen circulation [15]. The Eurasian golden jackal (*Canis aureus*) shows remarkable behavioral adaptability, thriving in habitats ranging from wilderness to agricultural landscapes and urban areas [16]. The golden jackal is currently recolonizing Europe, expanding from the Southeast across Central Europe into new territories in the North and the West. The Caucasus region has seen a rapid range expansion of the golden jackal already in the past decades, and is assumed to serve as a main source for the species' further expansion [17]. In Armenia, golden jackals are increasingly inhabiting anthropogenic environments where they come into direct contact with livestock and domestic animals [18, 19].

Parasitological surveys have documented diverse tick assemblages on golden jackals, including *Ixodes ricinus*, *Rhipicephalus sanguineus,* and *Dermacentor* spp., all of which are known as vectors for TBPs [20, 21]. Molecular screenings of European and Asian jackal populations have revealed infections with *Babesia*, *Hepatozoon*, *Anaplasma,* and *Rickettsia* species [22, 23], confirming that jackals can carry pathogens that are relevant to both animal and human health. However, data on TBP prevalence in jackal populations are rare, particularly in understudied regions where baseline epidemiological data are lacking.

Armenia, located in Transcaucasia, is an ideal region for investigating the ecology of TBP in wild carnivores. The country’s landscape displays high elevational gradients (380–4095 m), creating marked environmental heterogeneity [24]. This generates corresponding variation in tick vector distributions, with thermophilic species dominating lowland agricultural areas and cold-tolerant species occupying montane habitats [11]. Land use is characterized by a mosaic of smallholder agriculture, pastoral grazing, and peri-urban development, creating abundant ecotones where wildlife, livestock, and human activities overlap [25]. The Golden jackal population, which had been reduced to a minimum in the 1950s–1960s, has grown at an explosive rate over the past decades, especially in lowland and mid-elevation zones [24].

In this study, we present the first molecular epidemiological survey of tick-borne pathogens in golden jackals from Armenia. We employed PCR followed by reverse line blot (RLB) hybridization to screen blood samples from wild-caught individuals across six provinces. The objectives of this study were to: (1) determine the prevalence of tick-borne bacterial and protistan pathogens in golden jackals across Armenian regions; (2) assess co-infection patterns; and (3) evaluate demographic and geographic associations with the infection status.

## Methods

### Study area and sample collection

**Fig. 1:**
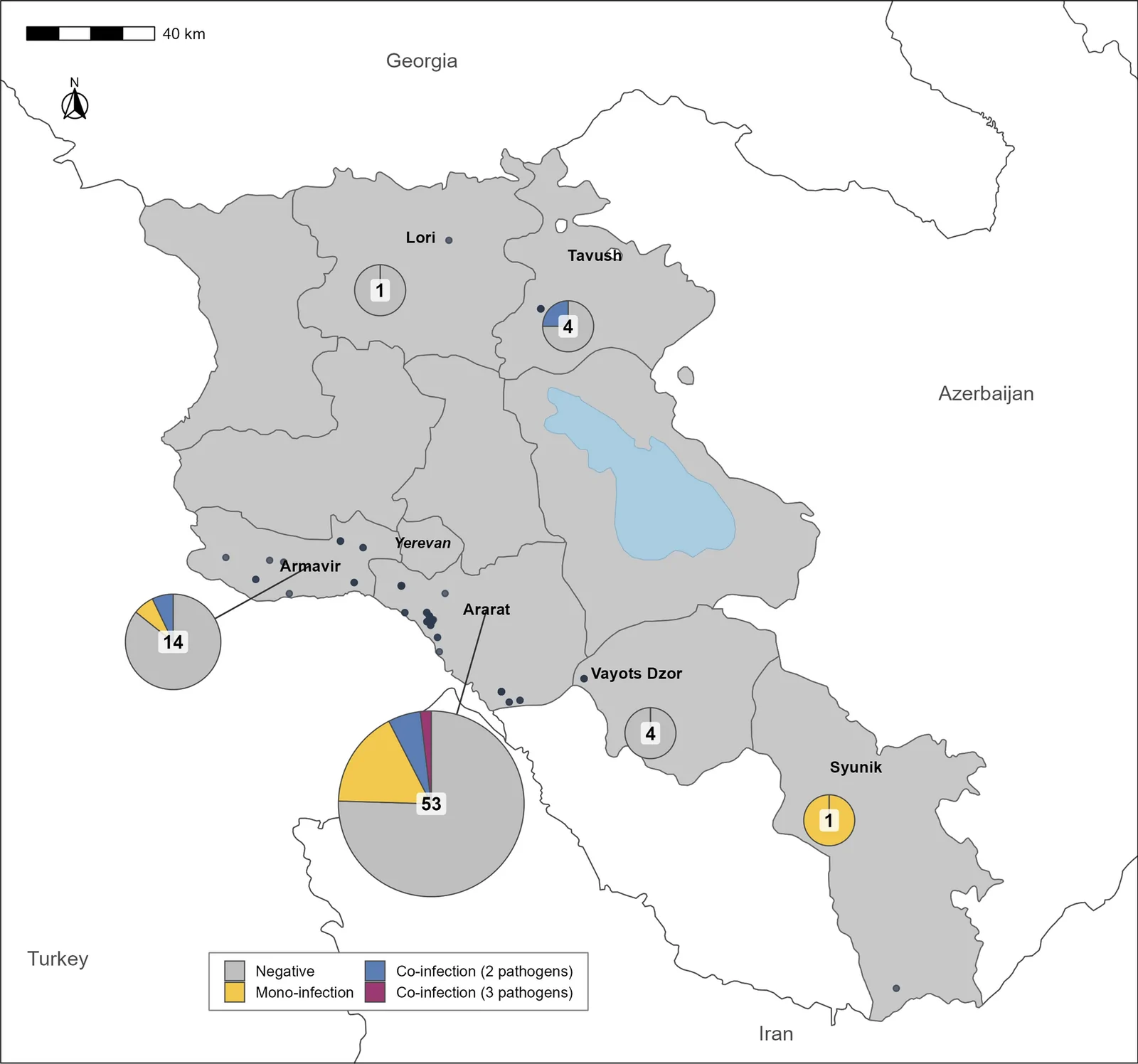
Geographic distribution of sampled golden jackals and infection status by province in Armenia. Dots indicate individual sampling locations. Pie charts display the proportion of negative (grey), mono-infected (yellow), co-infected with two pathogens (blue), and co-infected with three pathogens (magenta) animals per province. Numbers inside pie charts indicate the total number of jackals examined per province. Leader lines connect province labels to pie charts positioned outside the map for Ararat (*n* = 53) and Armavir (*n* = 14)

Between August 2024 and March 2026, blood samples were collected from 77 golden jackals from six administrative provinces of Armenia: Ararat (*n* = 53), Armavir (*n* = 14), Tavush (*n* = 4), Vayots Dzor (*n* = 4), Lori (*n* = 1), and Syunik (*n* = 1) (Fig. 1). Blood samples were collected during necropsy of jackals that were either road-killed or legally culled by local hunters during the authorized hunting season. Samples were preserved in DNA/RNA Shield (Zymo Research, USA; Cat. No. R1100-250) and transported to the Medical University of Vienna, Austria, for molecular analysis.

### DNA extraction

Total genomic DNA was extracted from 200 µL of blood using the QIAamp^®^ DNA Mini Kit (QIAGEN GmbH, Hilden, Germany) following the manufacturer's protocol for blood samples. DNA concentration and purity were assessed using a NanoDrop^™^spectrophotometer (Thermo Fisher Scientific, Vienna, Austria) and stored at − 20 °C until further use.

### PCR amplification

Genus-specific PCRs were performed, using 5’biotinylated reverse primers, as the first step to amplify pathogen DNA from blood samples, as previously described [26, 27]. Briefly, for *Anaplasma* and *Ehrlichia* spp., PCRs were targeting regions of the 16S rRNA gene, for *Rickettsia* spp., both the 16S rRNA gene and the 23S-5S intergenic spacer (IGS) region were amplified, and to detect *Borrelia* spp. DNA, the 5S-23S IGS was amplified. Additionally, the 18S rRNA gene was targeted for *Babesia/Theileria* spp. Primer sequences, target genes, and expected amplicon sizes are listed in Table 1.

**Table 1:** Primers used for PCR amplification of tick-borne pathogen DNA

Pathogen species	PCR region	Primer	Sequence (5' → 3')	Reference
*Borrelia burgdorferi* s.l	5S-23S IGS	F	ACCATAGACTCTTATTACTTTGACCA	[27]
		R	Biotin-GAGAGTAGGTTATTGCCAGGG	
*Rickettsia* spp.	16S rRNA	F	GAACGCTATCGGTATGCTAACA	[28, 29]
		R	Biotin-CATCACTCACGTTATGCTCTGA	
	23S-5S IGS	F	GATAGGTCRGRTGTGGAAGCAC	[30]
		R	Biotin-TCGGGAYGGGATCGTGTGTTTC	
*Anaplasma/Ehrlichia* spp.	16S rRNA	F	GGAATTCAGAGTTGGATCMTGGYTCAG	[31, 32]
		R	Biotin-CGGGATCCCGAGTTTGCCGGGACTTYTTCT	
*Babesia/Theileria* spp.	18S rRNA	F	GACACAGGGAGGTAGTGACAAG	[33]
		R	Biotin-CTAAGAATTTCACCTCTGACAGT	

*F* forward primer, *R* reverse primer

PCR reactions were performed in a total volume of 25 µL containing 5 µL of 5 × Phire Reaction Buffer, 0.5 µL of 10 mM dNTPs, 0.5 µL of each primer (20 µM), 0.125 µL of Phire Hot Start II DNA Polymerase (Thermo Fisher Scientific, Vienna, Austria), 2.5 µL of DNA template, and PCR-grade water (Sigma-Aldrich, Darmstadt, Germany) to final volume.

Thermal cycling conditions for *Anaplasma/Ehrlichia*, and *Rickettsia* 16S rRNA and *Babesia*/*Theileria* 18S rDNA consisted of initial denaturation at 98 °C for 30 s, followed by 10 cycles of 98 °C for 5 s, annealing from 67 to 57 °C (decreasing 1 °C per cycle) for 5 s, and extension at 72 °C for 7 s. This was followed by 45 cycles of 98 °C for 5 s, annealing at 57 °C for 5 s, and extension at 72 °C for 7 s, with a final extension at 72 °C for 1 min. For *Borrelia* spp., a similar touchdown program was used with an annealing temperature of 60 °C, which decreased at a rate of 1 °C per cycle to 50 °C during the first 10 cycles. Followed by 45 cycles with an annealing temperature of 50 °C. *Rickettsia* 23S-5S IGS was amplified using a similar touchdown PCR, with an initial annealing temperature of 65 °C and a decrease to 55 °C during the first 10 cycles. Afterwards, 45 cycles were carried out with an annealing temperature of 55 °C. Positive controls (pathogen DNA) and negative controls (PCR-grade water) were included in each PCR run to monitor amplification success and contamination.

PCR products were analyzed by gel electrophoresis on 1.5% agarose gels in 1 × TAE buffer, stained with GelRed Nucleic Acid Gel Stain (Biotrend, Cologne, Germany). Electrophoresis was performed at 100 V for 45 min, and bands were visualized using an iBright cl750 Imaging System (Thermo Fisher Scientific, Vienna, Austria) to confirm amplification and verify expected fragment sizes.

### Reverse line blot hybridization

Reverse line blot hybridization enables simultaneous detection and differentiation of numerous taxa with high sensitivity for low-level infections and mixed pathogen communities [34–36]. For simultaneous detection and differentiation of multiple tick-borne pathogens, RLB hybridization was performed following established protocols [26, 31, 35]. Species-specific oligonucleotide probes for *Anaplasma*, *Ehrlichia*, *Rickettsia*, *Borrelia*, *Babesia*, and *Theileria* species, along with genus-specific "catch-all" probes covalently linked to a Biodyne C nylon membrane (Pall Laboratories, Crailsheim, Germany), were used [27].

Biotin-labeled PCR products from each sample were pooled and denatured at 99 °C for 10 min, then immediately placed on ice. Denatured products were hybridized to the membrane at 42 °C for 60 min in 2 × SSPE (0.36 M NaCl, 20 mM NaH_2_PO_4_, 2 mM EDTA) containing 0.1% SDS using a miniblotter 45 (Immunetics, Boston, MA, USA). Following hybridization, membranes were subjected to stringent washing steps: two washes in 2 × SSPE/0.5% SDS at 50 °C for 10 min each, followed by a final wash in 2 × SSPE at room temperature for 5 min. Hybridized products were detected by incubating membranes with streptavidin–horseradish peroxidase conjugate (1:10000 dilution; Roche Diagnostics, Mannheim, Germany) for 30 min at 42 °C under gentle shaking, followed by detection after adding enhanced chemiluminescence substrate (ECL Western Blotting Substrate; Thermo Fisher Scientific, Vienna, Austria). Chemiluminescent signals were visualized using an iBright cl750 Imaging System. Samples producing signals with both genus-specific catch-all probes and species-specific probes were considered positive. Samples yielding only genus-specific signals without corresponding species-specific signals were subjected to sequencing for species determination.

### Statistical analysis

Descriptive statistics were used to calculate prevalence rates and 95% confidence intervals (CI) using the Clopper–Pearson exact method for each pathogen species. Associations between pathogen detection and host demographic variables (sex, age) were assessed using Fisher's exact test for categorical variables. Co-infection patterns were analyzed by constructing contingency tables comparing the presence or absence of different pathogen species. McNemar’s test was applied for pairwise comparisons of pathogens detected in the same individuals, and the tetrachoric correlation coefficient (*r*_tet_) was calculated to quantify these correlations. Statistical significance was set at *p* < 0.05. All statistical analyses were performed using R version 4.4.3 (R Core Team, 2024) with the base stats and polycor packages. Maps were created using QGIS (version 3.42.3) with base map data obtained from OpenStreetMap.

## Results

### Sample characteristics

Golden jackals examined (*n* = 77) included 36 females and 41 males, with 31 adults and 46 juveniles. The majority were from the Ararat (*n* = 53) and Armavir (*n* = 14) regions, with smaller numbers from Tavush (*n* = 4), Vayots Dzor (*n* = 4), Lori (*n* = 1), and Syunik (*n* = 1).

### Prevalence of tick-borne pathogens

*Rickettsia helvetica* was the most prevalent pathogen (*n* = 13), followed by *B. burgdorferi* s.s. (*n* = 3), *B. canis* (*n* = 3), *R. raoultii* (*n* = 2), *Ca.* N. mikurensis (*n* = 2) and *B. afzelii* (*n* = 1). Despite targeted screening via RLB hybridization, *Theileria* spp. and *Anaplasma* spp. were not detected in any sample (Table 2).

**Table 2:** Overall prevalence of tick-borne pathogens in golden jackals

Pathogen	Positive/total (77)	Prevalence (%)	95% CI*
*Rickettsia helvetica*	13	16.9	9.3–27.1
*Rickettsia raoultii*	2	2.6	0.3–9.1
*Borrelia burgdorferi* s.s	3	3.9	0.8–11.0
*Borrelia afzelii*	1	1.3	0.0–7.0
*Ca.* Neoehrlichia mikurensis	2	2.6	0.3–9.1
*Babesia canis*	3	3.9	0.8–11.0
*Anaplasma* spp.	0	0.0	0.0–4.7
*Theileria* spp.	0	0.0	0.0–4.7
Any pathogen	17	22.1	13.4–33.0

^*^CI confidence interval calculated using Clopper–Pearson exact method

### Co-infections

Co-infections were detected in six samples (representing 7.8% of all samples and 35.3% of pathogen-positive samples). One sample harbored three pathogens, *R. helvetica*, *Ca.* N. mikurensis, and *B. burgdorferi* s.s. Five jackals harbored co-infections: two with *R. helvetica* and *R. raoultii*, two with *R. helvetica* and *B. burgdorferi* s.s., and one with *B. canis* and *B. afzelii*. McNemar’s test revealed significant differences in prevalence between co-occurring pathogen pairs (*R. helvetica* vs *B. burgdorferi* s.s, *p* = 0.002; *R. helvetica* vs *R. raoultii,* *p* = 0.004). Tetrachoric correlation coefficients indicated strong positive co-occurrence between *R. helvetica* and *B. burgdorferi* s.s*.* (*r*_tet_ = 0.915), between *R. helvetica* and *R. raoultii* (*r*_tet_ = 0.878), and between *B. canis* and *B. afzelii* (*r*_tet_ = 0.955) (Fig. 1).

Overall pathogen prevalence was numerically higher in females compared to males. *Rickettsia helvetica* prevalence was higher in females (Fisher’s exact test, *p* = 0.030), and *B. burgdorferi* s.s. was detected exclusively in females, but this difference was not statistically significant (Table 3).

**Table 3:** Prevalence of tick-borne pathogens by sex and age

Pathogen	Female (*n* = 36)	Male (*n* = 41)	Adult (*n* = 31)	Juvenile (*n* = 46)
*Rickettsia helvetica*	10 (27.8%)	3 (7.3%)	8 (25.8%)	5 (10.9%)
*Rickettsia raoultii*	1 (2.8%)	1 (2.4%)	0 (0.0%)	2 (4.3%)
*Ca. N. mikurensis*	1 (2.8%)	1 (2.4%)	1 (3.2%)	1 (2.2%)
*Borrelia burgdorferi* s.s	3 (8.3%)	0 (0.0%)	3 (9.7%)	0 (0.0%)
*Borrelia afzelii*	1 (2.8%)	0 (0.0%)	1 (3.2%)	0 (0.0%)
*Babesia canis*	1 (2.8%)	2 (4.9%)	2 (6.5%)	1 (2.2%)
Any pathogen	11 (30.6%)	6 (14.6%)	10 (32.3%)	7 (15.2%)

Age-related comparisons showed higher overall infection rates in adults compared to juveniles. *Borrelia burgdorferi* s.s. and *B. afzelii* were detected only in adults. By contrast, *R. raoultii* was detected exclusively in juveniles. However, this difference was not statistically significant (Table 3).

### Geographic distribution of pathogens

The majority of infected animals originated from Ararat province (13/53; 24.5%), which harbored five of six detected pathogen species (*R. helvetica, R. raoultii, Ca. N. mikurensis, B. burgdorferi s.s., and B. canis*). In Armavir (*n* = 14), two animals (14.3%) tested positive with *R. helvetica*, *B. burgdorferi* s.s., and *B. canis*. One sample from Tavush (*n* = 4) was found co-infected with *B. canis* and *B. afzelii.* A single sample from Syunik was positive for *R. helvetica*. No pathogens were detected in samples from Vayots Dzor (*n* = 4) and Lori (*n* = 1) (Fig. 1). Due to geographic sampling imbalance, the regional comparison is not entirely reliable.

## Discussion

This study provides the first comprehensive molecular survey of tick-borne pathogens in golden jackals from Transcaucasia, documenting six zoonotic species with notable prevalences: *R. helvetica* (16.9%), *B. burgdorferi* s.s. (3.9%), *B. canis* (3.9%), *R. raoultii* (2.6%), *Ca.* N. mikurensis (2.6%) and *B. afzelii* (1.3%). The high frequency of co-infections (35.3% of pathogen-positive samples), including one triple co-infection, and the significant association between *R. helvetica* and *B. burgdorferi* s.s. (*p* = 0.002) and between *R. helvetica* and *R. raoultii* (*p* = 0.004) underscore the complexity of tick–pathogen–host interactions and highlight the epidemiological significance of golden jackals in maintaining regional transmission cycles.

### Regional context and novel findings

These are the first data on tick-borne pathogens in golden jackals from the Transcaucasia region, and they are in good agreement with the work by Aghayan et al. (2024) reporting an overall infection rate in Armenian tick populations of 64%, dominated by *A. phagocytophilum* (44%), *Theileria* spp. (36%), *Rickettsia* spp. (including *R. massiliae*), *B. burgdorferi* s.l.*, Francisella* spp., *Ca.* N. mikurensis, and *Babesia* spp. (~ 1%) [8]. In Georgia, positivity rates in tick pools have been reported to be 33% for *Rickettsia* spp. and 13% for *B. burgdorferi* s.l. [37, 38]. In southeastern Iran, *Rickettsia* spp. was detected in 24.9% of questing ticks [39]. *Rickettsia helvetica* has also been reported from Turkish tick populations, along with other spotted fever group *Rickettsia* spp., including *R. slovaca*, *R. massiliae*, and *R. monacensis* [28, 40]. The detection of *B. burgdorferi* s.s. in 3.9% of the investigated jackals provides the first confirmation of the circulation of the causative agent of Lyme borreliosis in wildlife in the Transcaucasus, complementing tick-based evidence from Armenia [8]. The documentation of *Ca.* N. mikurensis represents the first detection of this emerging human pathogen in golden jackals. Previous molecular surveys of wild canids have not detected this pathogen in red foxes from Bosnia and Herzegovina, north-eastern Italy, or Switzerland, despite using similar detection methods [41–43]. Rodents are considered the primary reservoir hosts for *Ca.* N. mikurensis, with *Ixodes ricinus* serving as the main vector. The detection of *Ca.* N. mikurensis in two jackals likely reflects individual spillover transmissions through shared tick vectors, as documented for other tick-borne pathogens in multi-host systems [44, 45].

### Pathogen-specific findings

The 16.9% *R. helvetica* prevalence detected in jackal blood is notable. Molecular detection of *Rickettsia* spp. in wild canid host blood or tissue has rarely been successful. Boretti et al. (2009) tested blood from 884 dogs and 58 foxes in Switzerland using a real-time TaqMan PCR assay specifically targeting *R. helvetica* without detecting any positive samples [46], and Millán et al. (2016) reported 0% *Rickettsia* in blood and spleen samples from 59 wild carnivores in Spain (red foxes, genets, stone martens, badgers), although *R. massiliae* was detected in ticks collected from the same animals by using conventional PCR [47]. The use of RLB hybridization in the present study, which offers high sensitivity for detecting low-level and mixed infections [34–36], may have contributed to the higher detection rate. The comparably high prevalence in Armenian jackals may reflect cumulative exposure through repeated tick infestations over the animal’s lifetime, as golden jackals occupy large ranges spanning diverse habitats. *Rickettsia helvetica* is increasingly recognized as an emerging human pathogen causing non-specific febrile illness and has been associated with perimyocarditis [48].

Two *B. burgdorferi* s.l. genospecies were detected: *B. burgdorferi* s.s*.* (3.9%) and *B. afzelii* (1.3%), both causative agents of Lyme borreliosis. These prevalences are comparable to those reported in other European wild carnivore populations. Hildebrand et al. (2022) documented 8.8% overall *Borrelia* spp. prevalence including *B. afzelii*, *B. garinii*, and *B. burgdorferi* s.s., in tissue samples from six mesocarnivore species (raccoon, raccoon dog, red fox, European badger, pine marten, stone marten) in Poland [49], and Hornok et al. (2013) detected *B. afzelii*, but not other *B. burgdorferi* s.l. genospecies in ticks collected from golden jackals in Hungary [34]. Interestingly, in the current study, *B. burgdorferi* s.s. was only detected in adult animals (9.7%), which may reflect cumulative exposure over a longer lifetime. *Borrelia afzelii* was detected in one jackal from Tavush province co-infected with *B. canis*. While carnivores are generally considered incompetent reservoir hosts for maintaining *B. burgdorferi* s.l. transmission cycles, they serve as sentinel species for pathogen circulation and human exposure risk [50]. All three *B. burgdorferi* s.s. positive samples also harbored *R. helvetica*, indicating a statistically significant co-infection pattern (*p* = 0.004) consistent with shared *Ixodes ricinus* ecology [51]. In Armenia, 6 confirmed cases of Lyme borreliosis had been documented in 2010 [52], and the current detection in jackals provides direct evidence of *Borrelia* circulation within a regional transmission system linking ticks, wildlife, and human populations. The identification of *R. raoultii* in 2.6% of the investigated jackals suggests active transmission involving *Dermacentor* ticks. *Rickettsia raoultii* may cause the so-called scalp eschar and neck lymphadenopathy after a tick bite (SENLAT) syndrome in humans [53].

The detection of *Ca.* N. mikurensis in two jackals (2.6%), one of them co-infected with both *R. helvetica* and *B. burgdorferi* s.s*.*, represents the first detection of this emerging pathogen in golden jackals and in Armenian wildlife. *Candidatus* N. mikurensis is an increasingly recognized cause of human neoehrlichiosis, particularly affecting immunocompromised individuals [44, 54].

*Babesia canis* was detected in three jackals (3.9%), representing the first detection in Armenian wildlife. Mitková et al. (2017) previously documented *B. canis* in golden jackals from Romania, demonstrating that expanding jackal populations may play a role in spreading and maintaining *B. canis* also into Europe [22]. Otranto et al. (2019) detected *Babesia* in 1.8% of golden jackals (1/55) and 5.3% of red foxes (2/38) from Iraq [55].

Neither *Anaplasma* spp. nor *Theileria* spp. were detected in the current golden jackal cohort. However, this is consistent with findings in other wild canid populations. Sgroi et al. (2021) found *A. phagocytophilum* in only 1.2% (3/244) of Italian foxes [56], suggesting that wild canids may not sustain detectable bacteremia for these pathogens. The absence of *Theileria* in jackal blood may reflect host tropism, as *Theileria* mainly infects ruminants rather than carnivores [57]. However, these results should be interpreted considering that RLB hybridization is more sensitive than conventional PCR and visualization using agarose gel electrophoresis but has a lower sensitivity than optimized quantitative real-time PCR for detecting low-burden infections. Aghayan et al. (2024) documented *Babesia* (~ 1%) and *Theileria* (36%) in Armenian ticks (*n* = 209) from domestic hosts and environmental samples, with *Theileria* prevalence showing significant geographic variation (*P* = 0.01) [8]. Regional data underline the medical relevance of these pathogens. Hosseini-Vasoukolaei et al. detected *A. phagocytophilum* in 25% (10/40) of human blood samples from shepherds in Mazandaran Province, Iran, predominantly affecting individuals older than 40 years with prolonged livestock contact [58], while *Babesia* was not detected in human samples despite prevalence in livestock in Lorestan Province, Iran [59].

### Co-infection dynamics and vector ecology

The high percentage of co-infections documented in this study is consistent with data from ticks in the region. Aghayan et al. (2024) reported that 31% of infected Armenian ticks harbored single pathogens, 27% harbored dual infections, and 6% harbored triple infections [8]. Similar co-infection patterns have been documented from adjacent regions. Studies in Iran have identified co-infections with *Anaplasma*, *Theileria*, and *Babesia* in both ticks and livestock [60, 61]. A Turkish study documented the co-occurrence of *Babesia*, *Theileria*, and *Anaplasma* in small ruminants [62], and a study from Georgia reported simultaneous detection of *Rickettsia* and *Borrelia* species in ticks [38]. These co-infections can result from either sequential acquisition through multiple tick bites or simultaneous pathogen exposure during a single feeding event [5]. *Ixodes ricinus* serves as the principal vector for *R. helvetica*, *B. burgdorferi* s.l., *Ca.* N. mikurensis, and *Anaplasma*/*Ehrlichia* species across Europe and Western Asia, facilitating simultaneous transmission of multiple pathogens [63, 64]. The co-infection with *B. canis* and *B. afzelii* in a single jackal from Tavush province is ecologically interesting and suggests co-exposure to both *Dermacentor* (vector for *B. canis*) [65] and *Ixodes* (vector for *B. afzelii*) [66] ticks in northern Armenia. In both, human and animal patients, co-infections may complicate clinical diagnosis and/or disease progression [67].

### Public health implications and study limitations

Our findings have direct relevance for zoonotic disease risk assessment in Armenia and the broader Transcaucasia. The detection of zoonotic pathogens at substantial prevalences in a widely distributed wild carnivore that frequently scavenges in peridomestic environments indicates active circulation of these pathogens in ecosystems where humans, livestock, and companion animals are exposed to shared tick populations [40]. Golden jackals increasingly inhabit agricultural landscapes and urban peripheries in Armenia, creating interfaces where pathogen spillover can occur.

Our study had several limitations. Although the sample size (*n* = 77) provided sufficient statistical power to detect a significant association between *R. helvetica* prevalence and host sex, the low number of positive animals for most pathogens (1–3 per species) limits the power for detecting further demographic associations. Also, the geographic sampling imbalance, with the majority of samples from Ararat province, prevents robust regional comparisons. We tested blood samples only. Some pathogens preferentially localize to specific tissues. Moreover, our molecular screening focused on bacterial and protistan pathogens, while tick-borne viruses were not assessed. Finally, the opportunistic sampling approach may introduce spatial, temporal, or demographic biases.

## Conclusions

This study documents six tick-borne pathogen species in Armenian golden jackals, including the first detection of *Ca*. N. mikurensis, *B. canis,* and *B. afzelii* in Armenian wild canids. The significant association between *R. helvetica* and host sex (*p* = 0.030), the high co-infection rate among positive animals (35.3%) and pathogen co-occurrence patterns highlight the complexity of tick–pathogen–host interactions. Our findings underscore the need to incorporate data from wildlife screenings into epidemiological considerations for tick-borne pathogens. As golden jackal populations continue to expand, particularly in Southeastern Europe and the Middle East, and jackals increasingly scavenge in human settlements, they may serve as bridges between sylvatic and urban transmission cycles.

## Data Availability

Data supporting reported results are contained within the article.
